# Research on shifting process control of automatic transmission

**DOI:** 10.1038/s41598-022-17413-7

**Published:** 2022-07-29

**Authors:** Wujun Zou, Ye Wang, Chaojie Zhong, Zhenchuan Song, Shenlong Li

**Affiliations:** Tank Transmission National Defense Science and Technology Key Laboratory, China North Vehicle Research Institute, Beijing, 100072 China

**Keywords:** Electrical and electronic engineering, Mechanical engineering, Engineering

## Abstract

The shift quality of an automatic transmission directly affects the human-perceived comfort and the durability of the automatic transmission. In general, the inconsistency caused by manufacturing errors, life-cycle changes, or other changes in hydraulic characteristics are the main reason affecting the shift quality, which should be compensated by adaptive control in the shifting process. In this paper, we first provide an in-depth analysis of the relationship between proportional solenoid current, clutch pressure, speed and torque in the shifting process control. Then we propose two efficient adaptive control strategies for the torque phase and inertia phase, respectively. Both algorithms are tested and verified on a riot utility vehicle. The experimental results indicate that the adaptive control strategies proposed in this paper can effectively compensate the engine flare and the clutch tie-up of the torque phase, and keep the inertia phase within a proper time range.

## Introduction

In recent years, with the advancement of science and technology, vehicles equipped with automatic transmissions are favored by consumers for their advantages of simple operation and large torque. In order to improve the fuel economy of the vehicles, the demand for the number of automatic transmission gears is also increasing, which makes the software control more complex and increases the workload of calibration. When shifting between gear ratios, the transmission control unit (TCU) should synchronize the engagement of the on-coming clutch and the disengagement of the off-going clutch, this process is called clutch-to-clutch shift control^1^. In a clutch-to-clutch shift^2,3^, smoothness of the shifting requires timing coordination between control actions involving the on-coming as well as the off-going clutches^4^. When hydraulic pressure is not appropriately controlled, the clutch can be engaged so quickly that driver and passengers may feel a shock, or so slowly that it takes long time to complete the change of the gears^5^. The shift quality is directly related to the comfort of human perception and the durability of automatic transmissions. In general, the inconsistency caused by manufacturing errors, life-cycle changes, or other changes in hydraulic characteristics are the main reason affecting the shift quality. However, relying on manual calibration^6^ to ensure a good shift quality is labor-intensive and financially expensive, and it does not meet the requirements of market laws^7^.

Around the core issue of improving the shift quality of automatic transmissions, many researchers have focused on the application of adaptive control. Adaptive control can adjust the control parameters to synchronize with the variations of physical characteristics, so it can continuously improve the shift quality. Deok-Ho Kim et al.^8^ utilize the adaptive neuro-fuzzy inference system as a supervisor and design the adaptive compensation scheme based on the investigation on shift characteristics, while this method takes a long time to train intelligent supervisors using selected experimental data and the algorithm is sensitive to the training data. Literature ^9^ focuses on the establishment of the adaptive fuzzy iterative control strategy, which contains a double-input-and -double-output fuzzy logic controller and a discrete iterative method, for the filling process of wet clutches in AT. The implementation of this algorithm is complex and requires high controller hardware, many parameters involved in the algorithm mainly depend on empiricism. Jinrank and Seibum^10^ proposed a method of torque estimation using the clutch friction model to realize the adaptive control of the shifting process, but in the actual vehicle application, the process of establishing the model through the sensor feedback data is complicated, and the established model is not accurate enough. Literature^11^ proposes three adaptive compensation strategies based on the friction coefficient-related parameters of the clutch surface, which are essentially the same as literature^10^. They focus on theoretical analysis and do not describe the methods applied to actual vehicles. Literatures^12–14^ is devoted to compensate for the influence of build-to-build variations and life-cycle variations on shifting process by analyzing the variation of clutch pressure characteristics and quantitatively adjusting the clutch pressure, but they barely describe the current control process. In the actual control process, the solenoid valve current is the most critical part as the direct output variable of the controller, and the clutch pressure as an intermediate observation variable is affected by the variable coupling factor, which is difficult to quantitatively control.

Most of the adaptive control algorithms proposed in the literature are essentially based on the angle of pressure regulation and clutch friction model identification to control the output of the desired speed curve. However, limited by the accuracy of the collected data and the characteristics of the system itself, the adaptive control algorithm based on clutch friction model identification has poor performance. In addition, the hydraulic system is a typical time-delay nonlinear coupling system, and the adaptive control strategy based on the quantitative adjustment of pressure is often difficult to realize in practical application. In practical applications, the electronic control system of the automatic transmission directly controls the solenoid valve and realizes the indirect control of parameters such as pressure, torque, and speed. Considering vehicle cost and structural complexity, vehicles on the market usually do not have torque meters and pressure sensors installed, but only the necessary speed sensors. Therefore, this paper starts with the solenoid valve control current output by the controller, and directly analyzes the relationship between the current of the actuator and the output speed, while the clutch pressure is only used as an intermediate observation variable for auxiliary analysis. We integrate the deviation between the actual speed and the expected speed into the shift quality evaluation system. Then, the solenoid valve control current is adaptively adjusted in accordance with the deviation of the shift quality, so as to realize the automatic compensation of the engine flare and clutch tie-up of the torque phase and keep the inertia phase within a proper time range.

In this paper, our motivation is to compensate as efficiently as possible the effects of manufacturing errors, life-cycle changes, or other changes in hydraulic characteristics on the shift quality of automatic transmission with a limited controller cost, so that it can be widely used in the market. The main contributions of this paper can be summarized as follows.

1. For the first time, we provide a thorough analysis of the relationship between proportional solenoid valve current, clutch pressure, speed and torque during gear shifting using a self-developed 7-speed automatic transmission as a model.
2. We propose two efficient adaptive control strategies for the torque phase and inertia phase of the shifting process. Both of these control strategies are methods for adaptively adjusting the TCU output current curve by calculating the deviation of the shift quality.
3. The two adaptive control strategies are both tested and verified on a riot utility vehicle. The experimental results show that the adaptive control strategies proposed in this paper can effectively compensate the engine flare and the clutch tie-up of the torque phase, and keep the inertia phase within a proper time range.

The following organizational structure of this article is as follows: firstly, we conduct a detailed analysis of the shifting process, and then the corresponding adaptive control strategies are proposed for the torque phase and inertia phase in the shifting process. In the experimental part, we test and validate our proposed adaptive control algorithm with a riot utility vehicle as the experimental object. Finally, we give our conclusions in the last section.

## Shifting process analysis

### Modeling of shifting process

**Figure 1:**
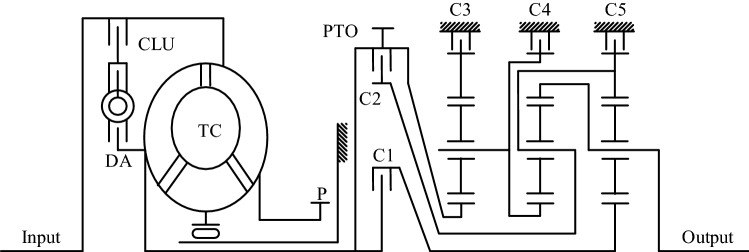
Scheme of 7-speed automatic transmission.

In order to analyze the dynamic characteristics of the shifting process, we first need to discuss the basic structure of an automatic transmission and its kinematics. In this paper, a completely domestic HPT 2006P automatic transmission designed by China North Vehicle Research Institute is selected as the research object. Figure 1 shows the scheme of the 7-speed automatic transmission, where Input represents the input shaft, Output represents the output shaft, DA is the shock absorber, TC is the torque converter, PTO is power take-out, P is oil pump, CLU is locking clutch, C1 and C2 are rotating clutches, C3, C4 and C5 are brakes.

As we can see, the automatic transmission can be divided into the power input module, the torque converter module, the planetary gear sets module and the power output module. The energy generated by the engine is transmitted from the power input module to the torque converter module, and then to the output module through the planetary gear sets module. Among them, the planetary gear ratio of different gears is the core part of the automatic transmission to realize the shifting operation, which is realized by the hydraulic system controlling the clutch and brake operating parts.

Hydraulic system consists of oil pump, oil pan and valve group. The oil pump is driven by the rotational speed of the input shaft and is the power source for the entire hydraulic system. The oil pan is covered with dense oil passages, and the flow direction, opening and closing and pressure of the oil in the oil passages are controlled by the valve group. The valve group is the direct control object of the automatic transmission controller, including the torque converter closing and unlocking valve CLU, the main pressure regulating valve, and the proportional valve and switching valve for gear control. Since the hydraulic system is a very complex system, in order to briefly explain the automatic transmission shifting process control concerned in this article, the following article mainly focuses on the proportional valve and switching valve of the shifting control.

Considering that the torque converter is relatively independent from the control of the shifting process, it can be assumed that the pump impeller and the turbine of the torque converter are mechanically connected during the shifting control process, that is to say the influence of hydraulic conditions on shift process control can be ignored. The control of the automatic transmission gear is realized by the hydraulic system controlling the pressure of the 5 clutches (i.e., C1, C2, C3, C4, C5), which in turn is determined by the combination of different states of the 2 proportional valves (i.e., PCS1, PCS2) and the 3 switching valves (i.e., SS1, SS2, SS3). The schedule of the corresponding relationship between the solenoid valve action and clutch pressure of each gear of the 7-speed automatic transmission is shown in Table 1. The “Y” in the table indicates that there is current output to the corresponding valve, and the white area indicates that the valve has no current. “●” indicates that the corresponding clutch has oil pressure, while the white area indicates that there is no oil pressure.

**Table 1:** Status of solenoid valve and pressure in each gear.

Gear	Solenoid valve					Clutch pressure					Ratio
	PCS1	PCS2	SS1	SS2	SS3	C1	C2	C3	C4	C5	
R		Y		Y	Y			●		●	− 5.0
N			Y	Y	Y					●	–
1				Y		●				●	3.5
2	Y	Y				●			●		1.89
3		Y				●		●			1.4
4	Y	Y	Y		Y	●	●				1.0
5					Y		●	●			0.73
6	Y	Y			Y		●		●		0.6

During the shifting process, one clutch is disengaged and the other is combined. Therefore, the force moment equilibrium calculations of the dynamic model can be expressed as follows.

The function of the engine-pump model include: the equivalent inertia of engine ${I}_{e}$, the engine speed ${W}_{e}$, the engine torque ${T}_{e}$ and the pump torque ${T}_{p}$.

1 $$\begin{array}{c}{I}_{e}\dot{{W}_{e}}={T}_{e}-{T}_{p}\end{array}$$

The function of the turbine-transmission shaft-off-going clutch model include: the equivalent inertia of turbine ${I}_{t}$, the turbine shaft speed ${W}_{t}$, turbine torque ${T}_{t}$, the input gear ratio ${i}_{in}$, the transmitted torque of on-coming clutch ${T}_{CL}$ and the transmitted torque of off-going clutch ${T}_{CH}$.

2 $$\begin{array}{c}{I}_{t}\dot{{W}_{t}}={T}_{t}-\frac{1}{{i}_{in}}\left({T}_{CL}+{T}_{CH}\right)\end{array}$$

The function of the on-coming clutch output shaft model include: the equivalent inertia of load ${I}_{v}$, the output shaft speed ${\omega }_{0}$, the output torque ${T}_{O}$, the equivalent torque of load ${T}_{v}$, the gear ratio before on-coming clutch ${i}_{ao}$ and the gear ratio before off-going clutch ${i}_{bo}$.

3 $$\begin{array}{c}{I}_{v}{\dot{\omega }}_{0}={T}_{O}-{T}_{v}={i}_{ao}\cdot {T}_{CL}+{i}_{bo}\cdot {T}_{CH}-{T}_{v}\end{array}$$

### Division of typical shifting types

A perfect shifting process has no power disturbances, and the transmission energy transfers smoothly between the on-coming clutch and the off-going clutch^15,16^. The research and test show that the input torque, speed, oil temperature and some factors that affect the characteristics of the hydraulic system are the main reasons that affect the shifting process^9,17^. Specific control laws can be formulated for different working conditions, and the control curves of different operating mode will also have obvious differences. Therefore, in order to meet the control requirements of various working conditions, it is necessary to classify the types of gear shifting process.

In general, there are four main shifting types, i.e., power on upshift, power on downshift, power off upshift, power off downshift. On the basis of the above, in the actual application, the shifting control process will be divided into more branches according to many factors affecting the characteristics of the shifting process, such as oil temperature, accelerator pedal position, slipping speed, engine torque and road conditions. The more branches, the wider the range of vehicle applications, but it will also increase the complexity of calibration parameters and control system. In addition, the change of characteristics in shifting process caused by mass production and life-cycle wear cannot be eliminated by adding branches. On the whole, it is very important to establish an appropriate number of branches and then take corresponding control strategies in accordance with different branches. Briefly, we introduce the main shifting types. Since power off downshift can in principle be controlled like power on upshift and power off upshift like power on downshift^13^, then we will analyze the power on upshift and power on downshift as an example.

**Figure 2:**
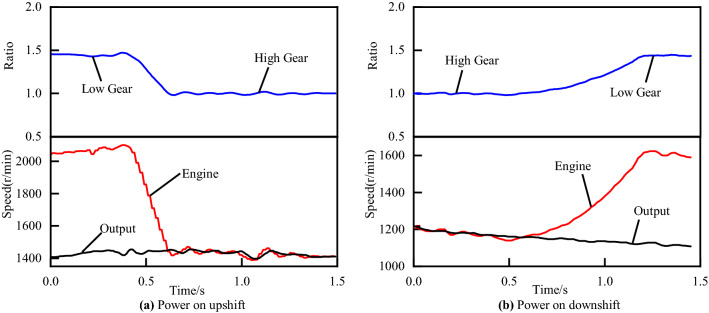
Shifting process of power on upshift and power on downshift.

Power on upshift occurs when the driver is driving the vehicle, and the speed increases continuously to reach the speed of shift point when stepping on the accelerator. Figure 2a shows a shifting process of power on upshift^18^. From the figure, we can see that the transmission ratio of the target gear in the power on upshift is less than that of the current gear. Therefore, in order to maintain a stable output speed before and after shifting, the input speed should logically decrease regularly. Power on downshift usually occurs when climbing a hill or resistance increasing^19^, the changes of speed and transmission ratio are shown in the Fig. 2b. Different from power on upshift, the engine speed increases during power on downshift to keep the output speed stable.

In essence, the shifting process control of automatic transmission is to control its speed to change according to the expected track. In fact, the principle of controlling the speed of the automatic transmission is closely related to the phase of shifting process, which will be introduced in the next section.

### Phase of shifting process

The shifting process takes only a small part of the whole working process, but it is the most important part. The shifting process control of automatic transmission depends on electro-hydraulic control system, which is composed of two parts: electronic control system and hydraulic actuation system. Consequently, we will analyze the shifting process from these two aspects^20^.

**Figure 3:**
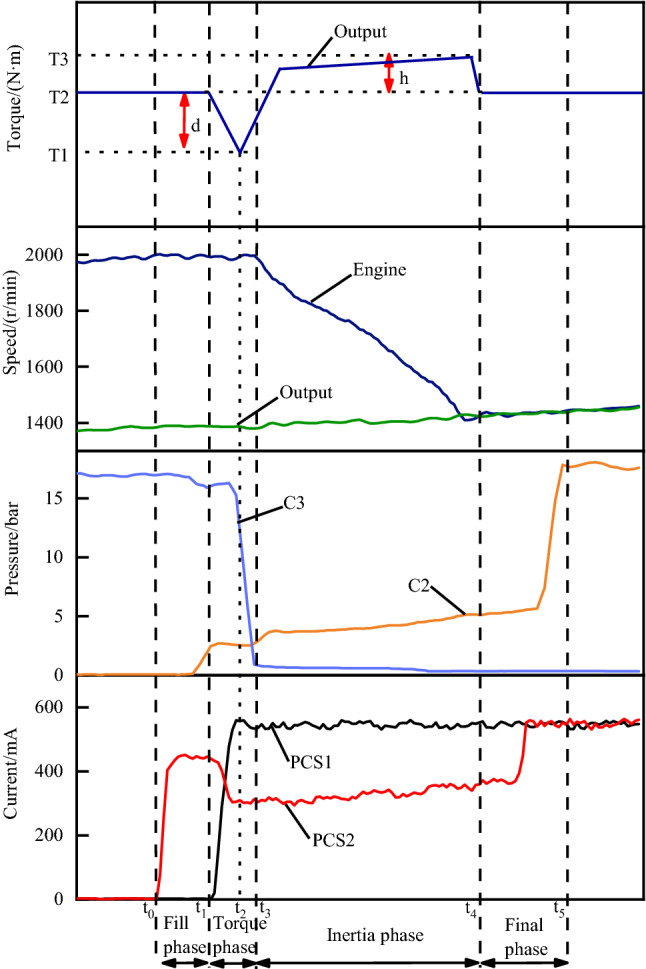
Phase of shifting process.

For example, the variation law of parameters in a typical upshift control process is shown in Fig. 3, which includes the variation curves of 4 parameters (i.e., proportional solenoid valve current, clutch pressure, engine speed and output speed, and the output torque) that we are mainly concerned about in the shifting process. The proportional solenoid valve current is directly output by the TCU, and the clutch pressure is a key intermediate process variable in the shifting control process. Due to the influence of the controller operation step size and the physical characteristics of the hydraulic system, the delay from the control current output to the clutch pressure response is about 30 ms. The quality of a shifting process is reflected in the changing laws of rotational speed and torque, but it is usually difficult to measure torque in actual vehicles, so we only present the theoretical output torque curve as auxiliary knowledge. In the process of shifting control, we usually divide it into four stages for control. These four stages are closely related and are also called four phases (i.e., fill phase, torque phase, inertia phase and final phase). Next, we will introduce these four phases respectively.

#### Fill phase

As shown in Fig. 3, at time ${t}_{0}$ when the shift command is generated, the fill phase occurs first, which firstly opens the filling solenoid valve (PCS2) to a large opening to make the on-coming clutch chamber quickly filled with oil before the clutch pressure starts to increase, this is the so-called kiss point. If the duration of fill phase is too long or the filling solenoid valve opens too much, the transmission will be damaged by premature engagement of the on-coming clutch, which is known as double gear.

#### Torque phase

At time ${t}_{1}$, the engine torque ${T}_{e}$ is transformed from the off-going clutch to the on-coming clutch. During this phase, the engine speed remains stable, with the current of drain solenoid valve (PCS1, normally closed valve) increases rapidly and the current of filling solenoid valve is reduced to a certain value to maintain the engaged clutch in the slip mode state. The slippage of the on-coming clutch and off-going clutch will consume the engine energy with an increase in oil temperature. As a result, the output torque decreases until the time ${t}_{2}$ where the off-going clutch transformed torque ${T}_{CH}$ is reduced to zero and the on-coming clutch transformed torque ${T}_{CL}$ equals the engine torque ${T}_{e}$. The phenomenon in which the output torque drops and then rises due to the overlap of torque during this period is also known as torque hole. Since the change in the output torque ${T}_{0}$ will directly reflect the change in the acceleration of the output speed, it is necessary to optimize the gradient of the torque hole curve. The control methods proposed in some literatures^14^, in order to obtain better shifting comfort, first reduce the pressure of the drain clutch to a certain point to prepare it to start the slippage. However, this also results in prolonged slippage, increased clutch wear and reduced service life. In this paper, we quickly reduce the pressure of off-going clutch without pause, and focus on the control of on-coming clutch pressure to get the appropriate point.

#### Inertia phase

At time ${t}_{3}$, the turbine speed ${N}_{t}$ begins to decrease, marking the end of torque phase and the beginning of the inertia phase as shown in Fig. 3. The force exerted on the on-coming clutch is increasing with the pressure continues to increase. This corresponds to an increase in the load. As a result, as the turbine speed ${N}_{t}$ decreases, the engine torque ${T}_{e}$ increases. If the pressure of on-coming clutch increase too quickly, the turbine speed ${N}_{t}$ and output torque ${T}_{0}$ will have a larger slope and will cause an uncomfortable ride. On the contrary, if the pressure of on-coming clutch increase too slowly, which will result in longer shift time.

#### Final phase

At time ${t}_{4}$, the turbine speed ${N}_{t}$ reaches the speed corresponding to the target gear, marking the end of the inertia phase and the beginning of the final phase. Since the on-coming clutch is no longer slipping at such point, the output torque is reduced to a position equivalent to the original $\mathrm{T}2$, then final phase happens. During this phase, the current of the filling solenoid valve is rapidly increased to reach the maximum value, so that the on-coming clutch is completely engaged, the slippage is stopped, and the entire shifting process is completed.

## Adaptive control strategy of shifting process

### Shift quality of shifting process

Shifting from a current forward speed ratio to a desired forward speed ratio requires that the clutching device associated with the current speed ratio be disengagement and the clutching device associated with the desired speed ratio be engagement. In its basic form, the control system directs the supply of fluid pressure to the transmission clutching devices in accordance with the control law table derived from empiricism. However, engine and transmission operating characteristics do change with time, and the production assembly tolerances may result in significant vehicle-to-vehicle variability. As a result, control schedules that produce acceptable ratio shifting in one vehicle may produce unacceptable ratio shifting in another vehicle. Hence, the controller needs to develop corrections for the empirically derived schedules involved in the shifting process so that when the shifting process is repeated at a later point, it will be performed in a more nearly optimum manner.

**Figure 4:**
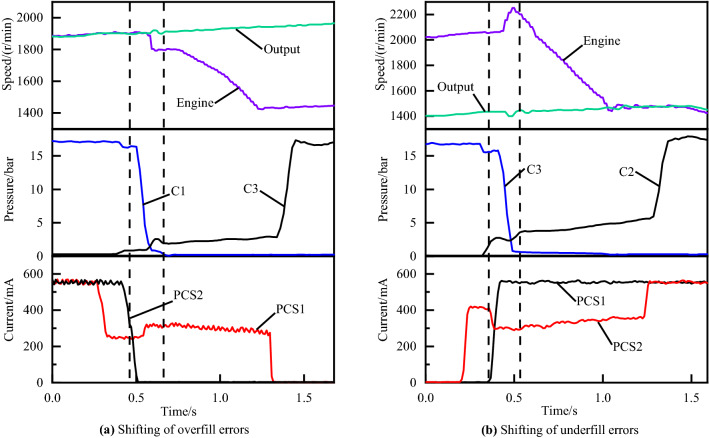
Shifting of underfill and overfill errors.

**Figure 5:**
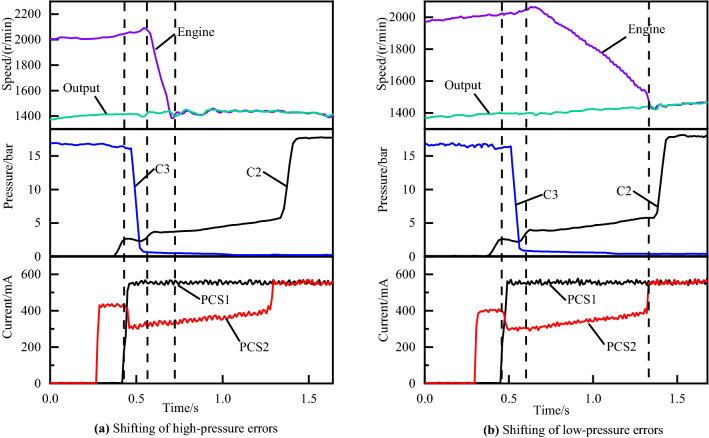
Shifting of low-pressure and high-pressure errors.

As explained below, the timing of such disengagement of off-going clutch and engagement of on-coming clutch is critical to the attainment of high-quality shifting process. If the on-coming clutch begins developing torque capacity with inappropriate fill time, the exchange of torque capacity between the off-going and on-coming clutch will not proceed according to schedule. In this regard, the graph of underfill errors and overfill errors can be illustrated as Fig. 4a,b, respectively. Similarly, the graph of the inertia phase duration errors which is too short or too long are illustrated in Fig. 5a,b respectively.

Figure 4a is a 60 percent throttle gear 4 to gear 5 power on upshift with overfill errors. Figure 4b is a 50 percent throttle 3–4 power on upshift with underfill errors. Figure 5a,b are gear 3 to gear 4 power on upshifts at 30 and 80 percent throttles with high-pressure and low-pressure errors, respectively.

In order to evaluate the quality of a shifting process and decide whether to adopt a compensation strategy^21^, literature^22^ summarizes all the indicator parameters of the shift quality, the expressions are as follows.

4 $$\begin{array}{l}{Q}_{s}=\left[ {J}_{s}, {T}_{s}, {\omega }_{s}, {\delta }_{s}\right]\end{array}$$

where ${Q}_{s}$ is the shift quality, ${J}_{s}$ is the rate of change of the transmission output torque which can be expressed as Eq. (5), ${T}_{s}$ is the time for transmission between two gears, ${\omega }_{s}$ is transmission output angular velocity deviation, ${\delta }_{s}$ is angular acceleration change rate.

5 $$\begin{array}{l}{J}_{s}=\frac{d{T}_{0}(t)}{dt}\end{array}$$

${\delta }_{s}$ can be expressed as Eq. (6)

6 $$\begin{array}{c}{\delta }_{s}=\frac{d\theta (t)}{dt}=\frac{s+1}{{r}_{w}}\frac{da(t)}{dt}\end{array}$$

where $\theta (t)$ is the angular acceleration of transmission output shaft, ${r}_{w}$ is tire radius, $s$ is constant tire slip, $a(t)$ is the linear acceleration of vehicle.

The vehicle driving dynamics equation is expressed as Eq. (7).

7 $$\begin{array}{c}{F}_{t}={F}_{f}+{F}_{w}+{F}_{i}+{F}_{j}\end{array}$$

${F}_{t}$ represents the tangential reaction force of the ground acting on the driving wheel, its expression is shown in Eq. (8). Where ${T}_{0}$ is transmission output torque, ${i}_{0}$ is main reduction gear ratio, ${\eta }_{0}$ is the drivetrain mechanical efficiency from transmission output to wheels.

8 $$\begin{array}{c}{F}_{t}={T}_{0}{i}_{0}{\eta }_{0}\end{array}$$

${F}_{f}$ represents the rolling resistance and its expression is shown in Eq. (9). Where $m$ is the vehicle weight, $g$ is the acceleration of gravity, $f$ is the coefficient of rolling resistance, $\alpha$ is the ramp angle.

9 $$\begin{array}{c}{F}_{f}=mgfcos\alpha \end{array}$$

${F}_{w}$ represents the wind resistance, its expression is shown in Eq. (10). Where ${C}_{D}$ is the air drag coefficient, $A$ is the windward area of vehicle driving direction, $u$ is the velocity of air relative to the vehicle.

10 $$\begin{array}{c}{F}_{w}=\frac{{C}_{D}A{u}^{2}}{21.15}\end{array}$$

${F}_{i}$ represents ramp resistance, its expression is shown in Eq. (11).

11 $$\begin{array}{c}{F}_{i}=mgsin\alpha \end{array}$$

${F}_{j}$ represents vehicle acceleration resistance, its expression is shown in Eq. (12). $\delta$ is the conversion factor of the rotating mass of the car after taking into account the moment of inertia of the rotating mass.

12 $$\begin{array}{c}{F}_{j}=\delta m\frac{du}{dt}\end{array}$$

Substituting Eqs. (8), (9), (10), (11), (12) into Eq. (7).

13 $$\begin{array}{c}{T}_{0}{i}_{0}{\eta }_{0}=mgfcos\alpha +\frac{{C}_{D}A{u}^{2}}{21.15}+mgsin\alpha +\delta m\frac{du}{dt}\end{array}$$

Differentiating Eq. (13), we can get Eq. (14).

14 $$\begin{array}{c}\frac{d{T}_{0}(t)}{dt}=\frac{1}{{i}_{0}{\eta }_{0}}\left(\frac{2{C}_{D}Au(t)a(t)}{21.15}+\delta m\frac{da(t)}{dt}\right)\end{array}$$

From Eq. (14), we can see that when the vehicle speed and longitudinal acceleration are relatively small, the rate of change of ${T}_{0}(t)$ is almost linearly related to the rate of change of $a(t)$, while the rate of change of ${T}_{0}(t)$ has more effect on the rate of change of $a(t)$ when the vehicle speed and longitudinal acceleration are increasing. This is consistent with the vehicle jerk perceived by the passengers on real vehicle.

Using above results, and ignoring the influence of the wind resistance sub-item on the shifting quality, the expressions of the shift quality defined as Eq. (15).

15 $$\begin{array}{c}{Q}_{s}={q}_{1}\left|{T}_{s}-{T}_{best}\right|+{q}_{2}\int \left|\omega (t)-{\omega }_{0}(t)\right|dt+{q}_{3}\int \left|\frac{da(t)}{dt}\right|dt\end{array}$$

where ${q}_{1}$, ${q}_{2}$, ${q}_{3}$, represent the weight coefficients of sub-items, $\omega (t)$ represents the actual speed, ${\omega }_{0}(t)$ represents the desired speed, ${T}_{best}$ denotes the optimal time determined by empiricism.

### Adaptive control strategy for the torque phase

Figure 4 shows the clutch tie-up curve and the engine flare curve, both of which, although occurring during the torque phase, are caused by inappropriate filling time. The filling time is usually not constant because of the engine and transmission operating characteristics changing with vehicle-to-vehicle variability and wear of the clutch during the life cycle. The use of manual calibration methods for filling time to compensate for this variation is not applicable to the market.

**Figure 6:**
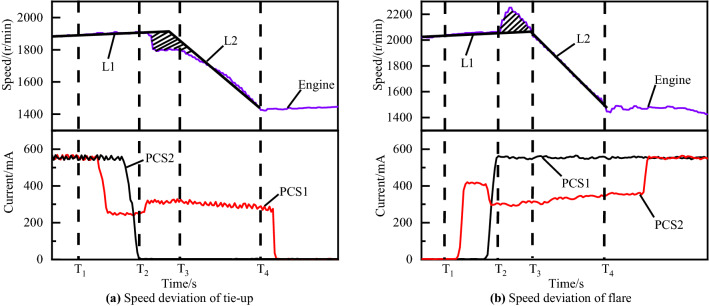
Principle of speed deviation for the torque phase.

In order to adaptively adjust the filling time, we need to calculate the speed deviation of the torque phase and determine whether it is engine flare or clutch tie-up. Figure 6 shows the calculation principle of the input speed deviation degree with clutch tie-up and engine flare.

As showing in Fig. 6, the calculation principle of the speed deviation of clutch tie-up and engine flare is basically the same, and the key is the establishment of the desired speed curve. The expected speed curve consists of two linear fitting curves L1 and L2. L1 is a curve fitted linearly by the engine speed between T_1_ and T_2_, and L2 is a curve fitted linearly by the engine speed between T_3_ and T_4_. Taking into account the time-delay characteristics of the actual solenoid valve control command and hydraulic system response, the rules for selecting the time points of the fitting time period are as follows: T_1_ is the point in time when a shifting request is generated and the shifting starts; T_2_ is the time point when the oil drain solenoid valve is fully opened; T_3_ equals T_2_ plus torque phase time(In this paper the torque phase time $\Delta {T}_{torque}$ is 0.2 s) as shown in Eq. (16); T_4_ is the time point when the transmission input speed reaches the target gear speed.

16 $$\begin{array}{c}{T}_{3}={T}_{2}+{\Delta T}_{torque}\end{array}$$

Assuming that the desired speed curve ${X}_{L}$ composed of L1 and L2 is L, its discrete expression is shown in Eq. (17). The discrete expression of the line segment representing the actual acquisition speed is shown in Eq. (18). The discrete expression of the line segment representing the output speed is shown in Eq. (19).

17 $$\begin{array}{c}{X}_{L}=\left\{{x}_{l}\left(0\right), {x}_{l}\left(1\right),{x}_{l}\left(2\right),\dots ,{x}_{l}(k)\right\}\end{array}$$

18 $$\begin{array}{c}{X}_{W}=\left\{{x}_{w}\left(0\right), {x}_{w}\left(1\right),{x}_{w}\left(2\right),\dots ,{x}_{w}(k)\right\}\end{array}$$

19 $$\begin{array}{c}{X}_{out}=\left\{{x}_{out}\left(0\right), {x}_{out}\left(1\right),{x}_{out}\left(2\right),\dots ,{x}_{out}(k)\right\}\end{array}$$

From Eqs. (17), (18) and (19), the discrete expressions for the integral of velocity deviation area ${A}_{rea}$ and acceleration rate of change ${B}_{acce}$ can be written as Eqs. (20) and (21). $\Delta \mathrm{t}$ is the sampling time interval.

20 $$\begin{array}{c}{A}_{rea}=\sum_{i=0}^{k}\left|{x}_{l}\left(i\right)-{x}_{w}(i)\right|*\Delta t\end{array}$$

21 $$\begin{array}{c}{B}_{acce}=\sum_{i=2}^{k}\left|\frac{{x}_{out}\left(i\right)-2{x}_{out}\left(i-1\right)+{x}_{out}(i-2)}{\Delta t}\right|\end{array}$$

**Figure 7:**
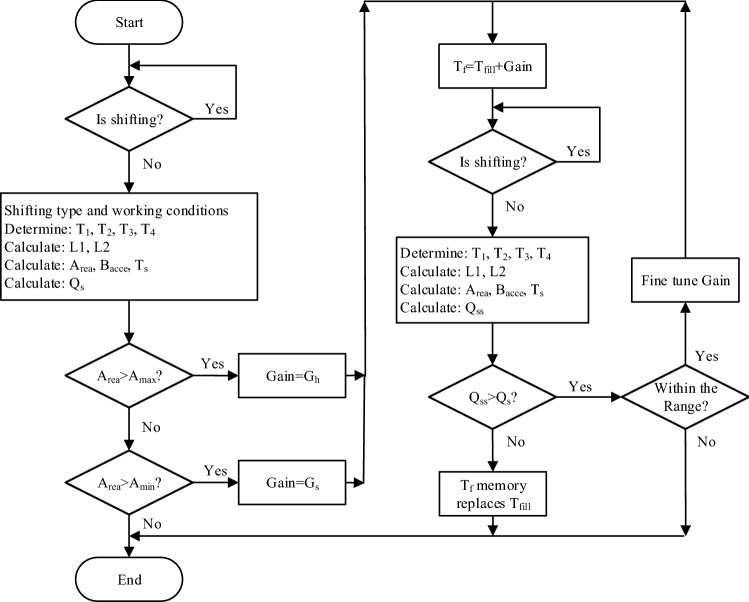
Flow chart of torque phase shift adaptation.

Figure 7 shows the flow chart of torque phase shift adaptation. In order to quickly and efficiently adjust the oil filling time adaptively, it is necessary to first judge the type of shifting process, calculate the speed deviation area, and then determine the value of the adaptive gain according to the speed deviation area. The adaptive gain will correct the initial value of the filling time. The new updated value will be stored in the memory for the corresponding shifting control parameters under the same working conditions if its shift quality is better than before. In addition, ${\mathrm{G}}_{h}$ and ${\mathrm{G}}_{s}$ in the Fig. 7 are positive when the shifting is clutch tie-up, while it is negative when the shifting is engine flare. ${Q}_{ss}$ is an indicator that is different from the original calculated ${\mathrm{Q}}_{s}$, and its calculation method is the same as that of ${\mathrm{Q}}_{s}$. ${\mathrm{A}}_{max}$ and ${\mathrm{A}}_{min}$ are the maximum and minimum values of the torque phase velocity deviation area threshold, respectively. Their values are obtained through experiments and manual experience, and the magnitude of the values is closely related to the order ratio of the shifting process. Regarding the parameters ${G}_{h}$ and ${\mathrm{G}}_{s}$, in the torque phase shift adaptation, ${\mathrm{G}}_{h}$ represents the maximum gain value of the oil filling time, which is 3 times the control period in this paper, while ${\mathrm{G}}_{s}$ represents the minimum gain value of the oil filling time adjustment, and its value is the control period time 10 ms.

### Adaptive control strategy for the inertia phase

The electro-hydraulic control system of the transmission is a typical time-delay nonlinear system. In order to prevent the system from running out of control, only one parameter is adjusted at a time. In view of the important role of the torque phase, in the practical application of the adaptive strategy, the oil filling time that affects the torque phase is first adjusted, and then the inertia phase is adaptively adjusted when the oil filling time is adjusted to a better state.

**Figure 8:**
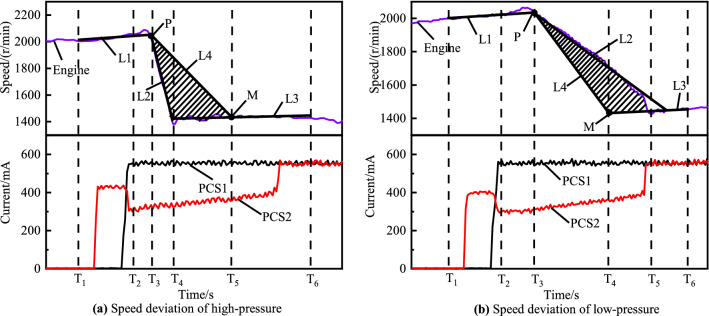
Principle of speed deviation for the inertia phase.

As showing in Fig. 8, the inertial phase adaptive strategy differs from the torque phase adaptive strategy in the choice of the desired speed profile for the calculation of the speed deviation. In this section the parameters ${\mathrm{T}}_{1}$, ${\mathrm{T}}_{2}$, ${\mathrm{T}}_{3}$, ${\mathrm{T}}_{4}$ are obtained in the same way as adaptive control strategy for the torque phase, the calculation method of ${\mathrm{T}}_{5}$ is shown in Eq. (22). ${\Delta T}_{inertia}$ is the optimal inertia phase duration, and its value is obtained experimentally and empirically. ${T}_{6}$ is the time when the final phase is completed.

22 $$\begin{array}{c}{T}_{5}={T}_{3}+{\Delta T}_{inertia}\end{array}$$

Then L1 can be obtained by linear fitting through the speed data between ${\mathrm{T}}_{1}$ and ${\mathrm{T}}_{2}$; L2 can be obtained by linear fitting through the speed data between ${\mathrm{T}}_{3}$ and ${\mathrm{T}}_{4}$; L3 can be obtained by linear fitting through the speed data between ${\mathrm{T}}_{4}$ and ${\mathrm{T}}_{6}$; point P is the intersection of lines L1 and L2; point M which can form L4 with point P can be determined by ${\mathrm{T}}_{5}$ and L3. Finally, we get the desired speed curve ${\mathrm{X}}_{L}$ composed of L1, L4, L3. The calculation method of parameters ${\mathrm{Q}}_{s}$, ${\mathrm{A}}_{rea}$, and ${\mathrm{B}}_{acce}$ is the same as that of the torque phase. It is worth noting that point P is the key point that determines the accuracy of the entire inertial phase adaptive algorithm, and it represents the theoretical working point where the torque phase ends.

**Figure 9:**
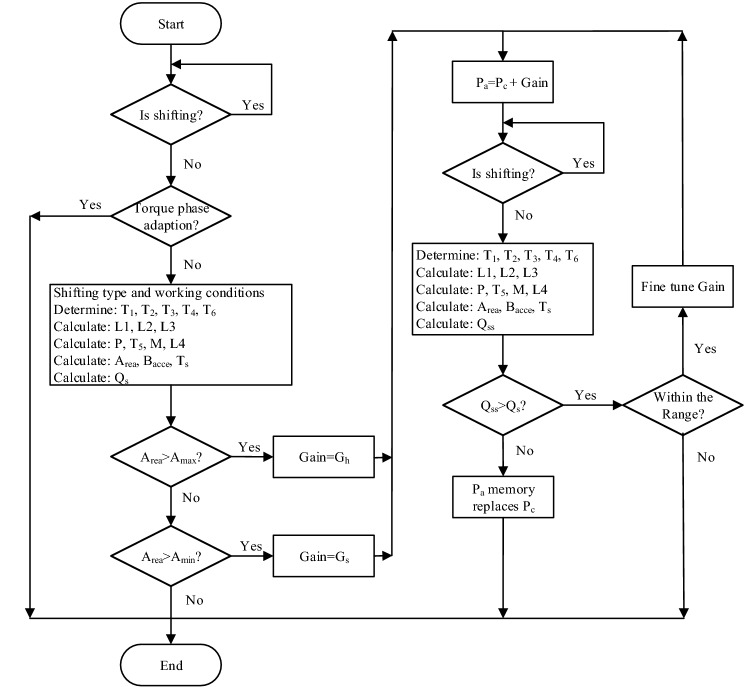
Flow chart of inertia phase shift adaptation.

Figure 9 shows the flow chart of inertia phase shift adaptation. Before executing the inertia phase adaptive control algorithm, first determine whether the torque phase adaptive control is currently performed. This is to avoid the control process system being out of control or unstable due to the simultaneous adjustment of the torque phase and inertia phase parameters. In the process of inertia phase adaptive control, the solenoid valve current is adaptively adjusted by calculating the deviation between the actual speed and the expected speed to adjust the pressure within a certain range. Finally, the shift quality is used to evaluate whether the shift process is optimized and decide whether to update the current parameters in memory. Where ${P}_{c}$ is the original solenoid valve current, and ${P}_{a}$ represents the solenoid valve current after adaptive adjustment.

In addition, it should be noted that in inertial phase shift adaptation, ${\mathrm{G}}_{h}$ represents the maximum gain value of current value adjustment, which is set to 30 in this paper, and ${\mathrm{G}}_{s}$ represents the minimum gain value of current value adjustment, which is set to 10 in this paper. If the value of ${G}_{h}$ is too large, the algorithm will not converge and become unstable; if the value of ${\mathrm{G}}_{s}$ is too large, the accuracy of the algorithm will deteriorate; and if the values of ${\mathrm{G}}_{h}$ and ${\mathrm{G}}_{s}$ are set too small, the algorithm will converge slowly and cannot meet the requirements of high efficiency.

## Vehicle experiment and analysis

**Figure 10:**
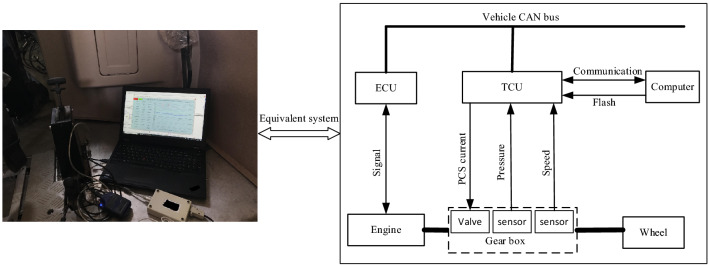
Experiment on riot utility vehicle.

The torque phase and inertia phase adaptive control strategies are tested and verified on a riot utility vehicle. Figure 10 is a picture of the real vehicle experiment, in which the real-time data acquisition and recording system is implemented by a self-developed software programmed by LabVIEW, and the automatic transmission control software incorporating the control strategy proposed in this paper is implemented in the TCU.

The core processor of the transmission control unit (TCU) is a high-performance 16-bit Freescale chip. In addition, in order to facilitate the analysis of shifting data during the entire driving process, in addition to installing the necessary speed sensors (including pump speed, turbine speed and output speed), 6 clutch pressure transducers are also installed. The sampling time of the TCU and the data acquisition and recording system are both set to 10 ms. Moreover, the detailed vehicle parameters and working conditions of the experimental vehicle used for adaptive control strategy testing are shown in Table 2.

**Table 2:** The vehicle parameters and working conditions.

Item	Conditions	Item	Conditions
Engine manufacturer	Dongfeng Cummins engine	Rear gear ratio	6.272
Engine type	ISDe300	Rear transmission efficiency	95%
Engine maximum power	220 kW	Wheel reducer speed ratio	3.478
Engine maximum torque	680 Nm	Tire radius	450 mm
Idling speed	800 rpm	Windward area	4.8m^2^
Oil temperature	80–95℃	Aerodynamic drag coefficient	0.75
Line pressure	10–20 bar	Rolling resistance coefficient	0.015
Road	Undulating concrete road	Vehicle mass	6.3t

**Figure 11:**
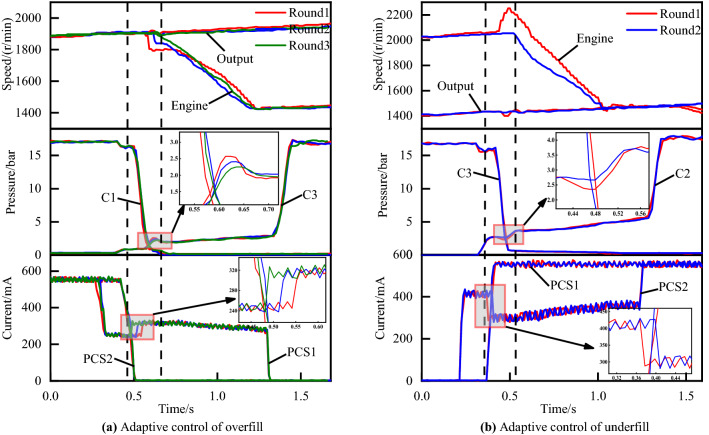
Adaptive control of overfill and underfill.

Figure 11a shows the experimental results of the adaptive control of overfill for the torque phase. In the figure, we can see that 3 sets of real vehicle experimental data are presented, corresponding to 3 adaptive adjustments for the filling time respectively. The red curve is the initial shifting control curve. The blue curve is the second test data after one adaptive compensation. The green curve is the final shifting control curve, representing the optimal shifting control curve after two adaptive compensation strategies. From the current graph and pressure graph, we can observe that the PCS1 solenoid valve filling time for round 3 is reduced by 60 ms compared to round 1, and the peak engaged clutch pressure C3 during the torque phase is reduced from 2.57 to 2.25 bar.

Figure 11b shows the experimental results of the adaptive control of underfill for the torque phase. The red curve is the shifting process curve before the adaptive adjustment. It can be seen from the engine speed curve that a flare phenomenon occurs in the torque phase. While a smooth shifting process occurs after adaptive tuning, in which the filling time of the solenoid valve PCS2 is increased by 30 ms compared with that before the adaptive adjustment, the corresponding valley value of the engaged clutch pressure C2 increases from 2.35 to 2.66 bar during the torque phase.

**Figure 12:**
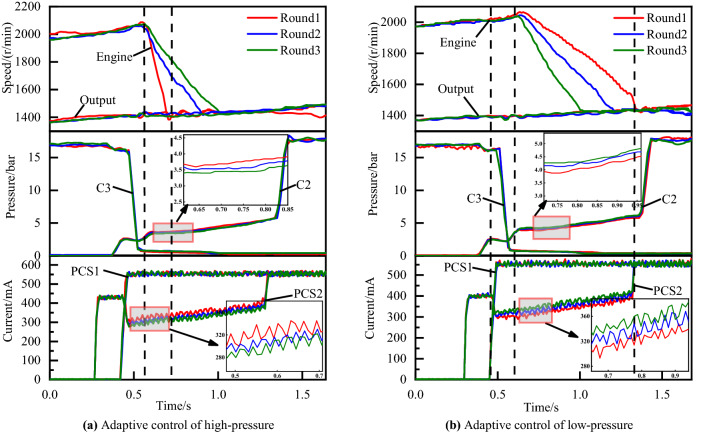
Adaptive control of high-pressure and low-pressure.

Figure 12 shows the vehicle experimental results of the gear 3 to gear 4 power on upshift with different inertia phase pressure adaptive control strategy. Figure 12a is the experimental result of inertia phase engaged clutch pressure C2 adaptive control under 30 percent throttle. It can be seen from the red engine speed curve that the C2 pressure during the inertia phase is high, it means that the engaging clutch receives more force and engages earlier, so the slope of the engine speed is larger. The adaptive control strategy will reduce the pressure of the engaged clutch in the inertia phase, as shown by the blue and green curves in Fig. 12a. The inertia phase duration of the optimal shift control curve after adaptive adjustment is increased from 141 to 458 ms.

Figure 12b is the experimental result of the adaptive control of inertia phase engagement clutch pressure C2 at 80% throttle. Different from the short inertia time error, when the long inertia time error occurs, the current of the engaged clutch solenoid valve PCS2 is adaptively adjusted to increase the inertia phase C3 pressure and make the collective clutch engage earlier. As shown in the engine speed curve in Fig. 12b, the inertia phase duration of the shifting after the optimization of the adaptive strategy is reduced from the original 736–409 ms.

Through the experimental results of the riot utility vehicle discussed above, the control parameters of the shifting process can be adaptively adjusted to the optimal range in the third round under normal circumstances, and can be adapted to a good value in the second round for some small deviations. Therefore, the efficiency of the adaptive control strategy is high enough to be applied to real vehicles.

## Conclusion

In this paper, we firstly use a self-developed 7-speed automatic transmission as a model to reveal the relationship between proportional solenoid valve current, clutch pressure, speed and torque in its shift control process. Considering the cost control requirements of market applications, the automatic transmission on the vehicle generally only installs the necessary speed sensor. Therefore, based on the premise of the solenoid valve control current, two adaptive control strategies are proposed for the torque phase and inertia phase for the purpose of improving passenger perceived comfort and component durability. The TCU software program incorporating the adaptive control strategy was developed and implemented on a riot utility vehicle equipped with a 7-speed automatic transmission. The experimental results indicate that the adaptive control strategy proposed in this paper can effectively compensate the engine flare and the clutch tie-up of the torque phase, and keep the inertia phase within a proper time range. The proposed shifting process adaptive control strategy can also be applied to other types of automatic transmissions to improve the influence of manufacturing errors, life-cycle changes, or other changes in hydraulic characteristics on shift quality.

## Data Availability

The datasets generated and analyzed during the current study are not publicly available due to involving the core parameters of our self-developed automatic transmission products but are available from the corresponding author on reasonable request.
